# Oral health of aboriginal people with kidney disease living in Central Australia

**DOI:** 10.1186/s12903-021-01415-4

**Published:** 2021-02-04

**Authors:** Kostas Kapellas, Jaquelyne T. Hughes, Alan Cass, Louise J. Maple-Brown, Michael R. Skilton, David Harris, Lisa M. Askie, Wendy Hoy, Basant Pawar, Kirsty McKenzie, Cherian T. Sajiv, Peter Arrow, Alex Brown, Lisa M. Jamieson

**Affiliations:** 1 Australian Research Centre for Population Oral Health, Adelaide Dental School, The University of Adelaide, Adelaide, Australia; 2 Wellbeing and Preventable Chronic Disease Division, Menzies School of Health Research, Charles Darwin University, Darwin, Australia; 3 Department of Nephrology, Division of Medicine, Royal Darwin Hospital, Darwin, Australia; 4 Boden Collaboration for Obesity, Nutrition, Exercise and Eating Disorders, Faculty of Medicine and Health, The University of Sydney, Sydney, Australia; 5 Department of Renal Medicine, Westmead Centre for Medical Research, Westmead Hospital, University of Sydney, Westmead, Australia; 6 NHMRC Clinical Trials Centre, University of Sydney, Sydney, Australia; 7 Griffith University, Brisbane, Australia; 8 NT Government, Darwin, Australia; 9 WA Government, Perth, Australia; 10 South Australian Health and Medical Research Institute, Adelaide, Australia

**Keywords:** Aboriginal Australian, Periodontal disease, Chronic kidney disease, End-stage kidney disease, Census, Population survey

## Abstract

**Background:**

Associations between kidney disease and periodontal disease are not well documented among Aboriginal people of Australia. The purpose of this investigation was to report and compare demographic, oral health, anthropometric and systemic health status of Aboriginal Australians with kidney disease and to compare against relevant Aboriginal Australians and Australian population estimates. This provides much needed evidence to inform dental health service provision policies for Aboriginal Australians with kidney disease.

**Methods:**

Sample frequencies and means were assessed in adults represented in six datasets including: (1) 102 Aboriginal Australians with kidney disease residing in Central Australia who participated in a detailed oral health assessment; (2) 312 Aboriginal participants of the Northern Territory’s PerioCardio study; (3) weighted estimates from 4775 participants from Australia’s National Survey of Adult Oral Health (NSAOH); (4) Australian 2016 Census (all Australians); (5) National Health Survey 2017–2018 (all Australians) and; (6) Australian Health Survey: Biomedical Results for Chronic Diseases, 2011–2012 (all Australians). Oral health status was described by periodontal disease and experience of dental caries (tooth decay). Statistically significant differences were determined via non-overlapping 95% confidence intervals.

**Results:**

Aboriginal Australians with kidney disease were significantly older, less likely to have a tertiary qualification or be employed compared with both PerioCardio study counterparts and NSAOH participants. Severe periodontitis was found in 54.3% of Aboriginal Australians with kidney disease, almost 20 times the 2.8% reported in NSAOH. A higher proportion of Aboriginal Australians with kidney disease had teeth with untreated caries and fewer dental restorations when compared to NSAOH participants. The extent of periodontal attachment loss and periodontal pocketing among Aboriginal Australians with kidney disease (51.0%, 21.4% respectively) was several magnitudes greater than PerioCardio study (22.0%, 12.3% respectively) and NSAOH (5.4%, 1.3% respectively) estimates.

**Conclusions:**

Aboriginal Australians with kidney disease exhibited more indicators of poorer oral health than both the general Australian population and a general Aboriginal population from Australia’s Northern Territory. It is imperative that management of oral health among Aboriginal Australians with kidney disease be included as part of their ongoing medical care.

## Background

Oral health is an important indicator of overall health and wellbeing [1]. Poor oral health can co-exist with many chronic conditions, such as diabetes and chronic kidney disease (CKD) [2–4]. Whilst the DMFT index provides an indicator of caries experience, its components can also be a useful proxy for access (or lack of access) to dental care and treatment received [5, 6].

Good oral health and provision of timely and appropriate dental services for Australians who have CKD and end-stage kidney disease (ESKD) is imperative, as pathology from the oral cavity contributes to overall inflammatory burden. Recent reports have shown the association of decline in kidney function and inflammation [7]. Furthermore, inflammation arising from periodontitis impedes glycaemic management in people who have diabetes [2]. Finally, optimal oral health which is free of infection is necessary for suitability for waitlisting for renal transplant [8]. Good oral health is especially essential for those undergoing renal transplantation, with transplantation inadvisable until all oral disease has been eradicated [9].

Both periodontal disease and untreated caries disproportionately affect Aboriginal Australians and people of lower socio-economic status [10]. Many reasons explain this inequity and include differences in access to affordable and timely dental care [11]. The maldistribution of dental practitioners to capital cities and major centres has a major impact on dental service availability and accessibility for those residing in regional or remote locations [12]. Nationally, 18% of Aboriginal Australians live in remote or very remote areas of Australia [13]. Although access to dental services in the Northern Territory is a priority for clients with dialysis-dependent ESKD across remote, regional and urban centres, this service need is often unmet.

The purpose of our study was to report demographic, oral health, anthropometric and systemic health status of Aboriginal Australians with kidney disease and to compare against relevant Aboriginal Australians and Australian population estimates. This new knowledge would inform current and future public health policy to mitigate oral health burden for Aboriginal Australians with kidney disease.

## Methods

Data describing oral health among Aboriginal people with and without kidney disease (102 and 312 respectively) and the general population were derived from detailed research studies in the Northern Territory, and from four nationally representative health datasets. The study involving Aboriginal adults with kidney disease was based in two Central Australian locations in partnership with both government and private dialysis units, and government dental clinics. Study participants comprised patients aged over 18 years who had a history of CKD, including ESKD requiring dialysis. The study aims were to describe the extent and severity of periodontal disease among a group of Aboriginal adults with CKD and to examine the effects of a comprehensive periodontal intervention among an Aboriginal population with CKD [14]. Thirty-two Aboriginal Australians with kidney disease did not undergo an oral assessment due to coagulation risk or feeling unwell during the data collection phase. This meant that sociodemographic data was available for 102 Aboriginal adults with CKD, while clinical dental data was available for 70. The first comparison was against 312 Aboriginal Australians without kidney disease who took part in the screening process for the PerioCardio study (based in Darwin, Katherine and Alice Springs). To be eligible for the PerioCardio study, participants needed to be aged 18+ years and to have periodontal disease (ascertained from the screening). The aims of the PerioCardio study were to describe the extent and severity of measures of vascular health and inflammation in Aboriginal Australians with moderate or severe periodontal disease, to determine if there was a dose–response relationship between extent and severity of periodontal disease and measures of vascular health and inflammation, and to determine whether periodontal treatment influences vascular health and inflammation among Aboriginal Australians with moderate to severe periodontal disease [15]. Sociodemographic data was available for 312 PerioCardio study participants, while clinical dental data was available for 273. Other population-level comparisons included: (1) weighted estimates from 4,775 National Survey of Adult Oral Health 2004–2006 (NSAOH) participants [16]; (2) 2016 Australian Census data [17]; (3) National Health Survey (NHS) 2017–2018 [18] and; (4) Australian Health Survey (AHS): Biomedical Results for Chronic Diseases, 2011–2012 [19]. Across all six datasets we examined demographic, oral health and biomedical estimates.

### Oral assessment

Oral assessments replicated the methods used in NSAOH [20]. Collected data included tooth presence, caries experience, periodontal destruction, gingival bleeding, dental plaque and calculus scores. A single caries experience score was recorded for each tooth using the following hierarchy: sound, filled due to caries, filled unsatisfactorily (defective restorations without decay), recurrent caries (new decay around existing filling) and decayed. Dental plaque and calculus were recorded for six teeth, which were the most anterior molar in each quadrant, in addition to the maxillary right central and mandibular left central incisors (if present) [21]. Periodontal probing depth (PPD) and gingival recession to calculate clinical attachment level (CAL) was measured at four sites of every tooth excluding third molars, which included the mesio-buccal, mid-buccal, disto-buccal and disto-palatal/lingual. ‘Moderate’ or ‘severe’ periodontal disease was determined by combining PPD and CAL based on the Centres for Disease Control and Prevention and the American Academy of Periodontology 2012 case definition [21]. Gingival bleeding on probing (BOP) was based on the established Gingival Index criteria [22].

### Other clinical measurements

Height was measured to the nearest 0.1 cm using a portable stadiometer (Seca 213, Hamburg, Germany). Weight was measured to the nearest 0.1 kg using a portable weight scale (Tanita HD-351, Arlington Heights, USA) with participants lightly clothed. Body mass index (BMI) was calculated as weight (kg) divided by the square of height (m). Systolic and diastolic blood pressure was measured using the SphygmoCor XCEL device that automates measurement using a brachial cuff via three one-minute cuff inflation cycles, and central blood pressure estimated by use of a proprietary general transfer function (AtCor Medical, Sydney). Bilateral carotid intima-media thickness was asssessed by high-resolution ultrasound from a single angle of convenience from both left and right carotid arteries in the PerioCardio study [15], and from up to 3 angles per carotid artery in the study involving Aboriginal Australians with kidney disease.

### Statistical analysis

All data were restricted to study participants aged between 18 and 80 years to reflect the age distribution in the sample of Aboriginal Australians with renal disease. Sample frequencies and means along with their respective 95% confidence intervals were generated to enable comparisons across datasets. Statistically significant differences were denoted by non-overlapping 95% confidence intervals (CIs). Whenever there is a lack of overlap between 95% CIs for two groups, it is a mathematical certainty that a hypothesis test of the difference between the same two groups would yield a P-value of less than 0.05. In order to calculate the standard deviation of mean values reported in both the NHS and AHS from the standard error, the following formula was used: SD $=SE \surd N$. To account for differences in the number of teeth between study samples, extent scores using the methods described by Carlos and colleagues [21] were generated to permit comparisons of CAL, PPD and BOP. All analyses were performed using SAS 9.4 (Cary, NC, USA).

## Results

Data were available for 102 Aboriginal Australians with renal disease; 85 with ESKD (83%) and 17 non-ESKD (17%). The average age was 48.1 years and the majority had achieved only up to a school education level (74.7%) (Table 1). When comparing against other population estimates, the average age of Aboriginal Australians with kidney disease was significantly higher than participants enrolled in the PerioCardio study (41.0 years) and NSAOH (40.0 years) (Table 1). A higher proportion of Aboriginal Australians with kidney disease had completed school-only education and were unemployed or not seeking work when compared to PerioCardio study estimates (total proportion 46.3%), NSAOH participants (total proportion 37.0%) or the Australian population (total proportion 35.7%). Most Aboriginal Australian participants with renal disease were unemployed (52.7%) or not seeking work (33.3%). The proportion of Aboriginal Australians with kidney disease who were current smokers was 38 percent. The proportion of Aboriginal Australians with kidney disease who were current smokers was half that of PerioCardio study participants. However, Aboriginal Australians with kidney disease were twice as likely to be current and half as likely to be former smokers compared to the Australian population and the NSAOH sample (Table 1).

**Table 1:** Socio-demographic characteristics of Aboriginal Australians with kidney disease in Central Australia compared with PerioCardio participants and other population estimates

	Aboriginal Australians with kidney disease in Central Australia % (95% CI) (N = 102)	PerioCardio study % (95% CI) (N = 312)	NSAOH 2004–2006% (95% CI) (N = 4775)	Australian population estimates^1,2^
Mean (SD) age*	48.11 (45.57, 50.65)^†^	40.01 (38.87, 41.15)^†^	44.84 (44.09, 45.59)	–
**Sex**^1^				
Female	60.8 (51.1, 70.4)^†^	44.2 (38.7, 49.8)^†^	50.3 (48.4, 52.1)	50.8
Male	39.2 (29.6, 48.9)^†^	55.8 (50.2, 61.3)^†^	49.7 (47.9, 51.6)	49.2
**Highest school level completed**^1^				
Nil/primary	30.3 (20.6, 40.1)^†^	13.1 (9.2, 17.1)^†^	2.3 (1.8, 2.8)^†^	0.9
Years 8/9	32.6 (22.7, 42.5)	20.8 (16.1, 25.5)	6.9 (6.0, 7.8)^†^	9.0
Years 10/11	22.5 (13.6, 31.3)^†^	42.6 (36.8, 48.3)^†^	30.8 (29.0, 32.7)	29.6
Year 12	14.6 (7.1, 22.1)^†^	23.5 (18.6, 28.4)	60.0 (57.9, 62.1)^†^	60.5
**Post-school qualification**^1^				
Nil/school only	74.7 (65.6, 83.8)^†^	50.3 (44.6, 56.1)^†^	36.8 (34.8, 38.8)^†^	42.2
Trade/apprenticeship	3.3 (0.0, 7.0)^†^	5.5 (2.9, 8.2)	14.1 (12.7, 15.4)^†^	30.4^b^
Certificate/diploma	18.7 (10.5, 26.8)^†^	33.4 (28.0, 38.9)^†^	12.4 (11.1,13.7)	
University degree	3.3 (0.0, 7.0)^†^	10.7 (7.1, 14.3)^†^	36.8 (34.6, 38.9)^†^	27.4
**Employment status**^**1**^				
CDEP**	3.2 (0.0, 6.9)	5.4 (2.7, 8.0)	–	–
Full-time	4.3 (0.1, 8.5)^†^	42.3 (36.5, 48.1)^†^	28.0 (11.4, 44.5)^†^	42.4
Part-time	6.5 (1.4, 11.5)	6.1 (3.3, 8.9)	35.0 (18.2, 51.8)	21.9
Unemployed	52.7 (42.4, 63.0)^†^	6.5 (3.6, 9.4)^†^	37.0 (19.9, 54.2)^a^	4.5
Not seeking work/retired/disability	33.3 (23.6, 43.1)	39.8 (34.0, 45.6)		31.2
**Main source of income**				
Government benefits	87.2 (80.4, 94.1)^†^	21.2 (16.4, 26.1)^†^	29.7 (27.8, 31.5)	–
Wages/salary	6.4 (1.3, 11.4)^†^	46.0 (40.1, 51.9)^†^	68.3 (66.5, 70.1)	–
Student	-	1.1 (0.0, 2.3)	2.0 (1.3, 2.7)	–
Other	6.4 (1.3, 11.4)^†^	31.7 (26.2, 37.2)^†^	–	–
**Smoking status**^**2**^				
Current	38.4 (27.9, 48.9)^†^	66.1 (60.2, 72.1)^†^	17.0 (15.5, 18.6)^†^	15.1 (14.6, 15.7)^†^
Former	12.8 (5.6, 20.0)^†^	10.6 (6.7, 14.5)	29.2 (27.6, 30.7)^†^	29.2 (28.5, 29.8)^†^
Never	48.8 (38.1, 59.6)^†^	23.3 (17.9, 28.6)^†^	53.8 (51.9, 55.7)	55.7 (55.0, 56.4)
**Chew tobacco**				
Yes	21.7 (13.2, 30.3)^†^	5.3 (2.7, 7.9)^†^	–	–
No	78.3 (69.7, 86.8)^†^	94.7 (92.1, 97.3)^†^	–	–
**Standard alcohol drinks/week**^**2**^				
None	56.6 (45.2, 68.0)^†^	36.4 (30.6, 42.2)^†^	-	43.9 (43.2, 44.7)^†^
1–7 drinks	27.6 (17.3, 37.9)	33.1 (27.5, 38.7)	–	38.9 (38.2, 39.6)
8–19 drinks	6.6 (0.9, 12.3)	11.0 (7.3, 14.8)	–	6.3 (6.0, 6.7)
20 or more	9.2 (2.6, 15.9)	19.5 (14.7, 24.2)	–	9.8 (9.4, 10.2)
**Periodontal case status#**				
Non-case or mild	7.1 (1.0, 13.3)^†^	12.6 (8.9, 16.3)	74.1 (72.3, 75.9)^†^	–
Moderate	38.6 (26.9, 50.3)^†^	60.6 (55.2, 66.1)^†^	23.1 (21.5, 24.7)^†^	–
Severe	54.3 (42.3, 66.2)^†^	26.8 (21.8, 31.7)^†^	2.8 (2.2, 3.4)^†^	–

^1^2016 Australian Census (limited to 18–80 years of age)

^2^National Health Survey First Results 2017–2018 (18 + years of age)

^*^Mean (95% CI) reported

^**^ Community Development Employment Project, a government-initiated employment scheme

^#^CDC-AAP 2007 case definition

^a^Proportion around 95% CI from PerioCardio study combines unemployed, not seeking work, retired, disability

^b^Proportion from 2016 Australian Census combines Trades, apprenticeship, certificate and vocational diploma

^†^denotes non-overlapping 95% confidence intervals

Of the 70 Aboriginal Australians with kidney disease who provided clinical oral health data, almost all (92.9%) had ‘moderate’ or ‘severe’ periodontitis. The estimate for ‘moderate’ or ‘severe’ periodontitis for Aboriginal Australians with kidney disease was significantly greater than the 25.9% in NSAOH. The prevalence of ‘severe’ periodontitis was two times higher among Aboriginal Australians with kidney disease (54.3%) compared with the PerioCardio study participants (26.8%) and almost 20 times higher than general Australian population estimates (2.8%).

The mean number of teeth with untreated caries among Aboriginal Australians with kidney disease was 3.2 which was five times higher than that reported for NSAOH participants (0.6), but not significantly different to estimates from the PerioCardio study (3.0) (Table 2). Just over half had periodontal clinical attachment loss (CAL) of 4 mm or more. The extent of sites with periodontal pocket depth (PPD) of 4 mm or more was 21.4%, while 31.9% experienced moderate or heavy bleeding on probing. Examining historical evidence of periodontal disease, Aboriginal Australians with kidney disease exhibited more than twice the number of sites with CAL ≥ 4 mm compared with PerioCardio participants (22.0%); this was tenfold higher than that of national survey estimates of the Australian population (5.4%). In reference to the presence of current disease, a two-fold difference in the extent of sites with PPD ≥ 4 mm and ‘moderate’/’heavy’ bleeding on probing was seen between Aboriginal Australians with kidney disease and PerioCardio study participants (12.3% and 16.0% respectively).

**Table 2:** Oral health parameters of Aboriginal Australians with kidney disease in Central Australia compared with PerioCardio participants and Australian population estimates

	Aboriginal Australians with kidney disease in Central Australia mean (95% CI) (N = 70)	PerioCardio study mean (95% CI) (N = 273)	NSAOH 2004–2006 mean (95% CI) (N = 4,775)
Number of teeth	24.34 (22.89, 25.79)^†^	26.50 (25.86, 27.14)^†^	25.59 (25.38, 25.79)
Number of decayed teeth	3.17 (2.47, 3.87)^†^	3.02 (2.64, 3.40)	0.59 (0.53, 0.66)^†^
Number of missing teeth	6.40 (4.92, 7.88)^†^	3.83 (3.22, 4.44)^†^	4.92 (4.68, 5.17)
Number of filled teeth	1.29 (0.81, 1.76)^†^	2.87 (2.46, 3.28)^†^	8.49 (8.21, 8.78)^†^
DMFT	10.86 (9.20, 12.51)^†^	9.72 (8.88, 10.56)	14.01 (13.60, 14.42)^†^
Mean pocket depth	2.82 (2.63, 3.01)^†^	2.32 (2.25, 2.38)^†^	–
Extent AL ≥ 4 mm (%)	51.03 (42.87, 59.19)^†^	21.96 (19.42, 24.50)^†^	5.37 (4.93, 5.82)^†^
Extent PPD ≥ 4 mm (%)	21.38 (16.14, 26.62)^†^	12.26 (10.74, 13.79)^†^	1.31 (1.03, 1.59)^†^
Extent PPD ≥ 6 mm (%)	3.48 (0.82, 6.14)	1.15 (0.82, 1.48)	–
Extent AL ≥ 3 mm & PPD ≥ 4 mm (%)	20.49 (15.41, 25.56)^†^	12.10 (10.59, 13.61)^†^	1.20 (1.02, 1.38)^†^
Extent index sites with calculus (%)^*^	88.61 (82.98, 94.24)	–	–
Extent index sites with ‘moderate’/ ‘heavy’ plaque (%)^*^	79.39 (70.65, 88.14)	–	–
Extent ‘moderate’/ ‘heavy’ BOP (%)	31.93 (21.45, 42.40)^†^	16.01 (13.94, 18.09)^†^	–

^*^six index sites examined

DMFT: mean number of decayed, missing and filled teeth

AL: attachment loss

PPD: periodontal pocket length

BOP: bleeding on probing

^†^denotes non-overlapping 95% confidence intervals

The mean systolic blood pressure of Aboriginal Australians with renal disease was 143 mmHg, significantly higher than estimates from both the general Australian population (123 mmHg) and from the PerioCardio study (125 mmHg) (Table 3). Mean HbA1c was higher among Aboriginal Australians with kidney disease (54.4 mmol/mol) compared with national estimates (36.2 mmol/mol; data on HbA1c in national samples stratified by diabetes not available), and estimates from the PerioCardio study (45.0 mmol/mol) although the 95% confidence intervals overlapped. The mean total cholesterol of Aboriginal Australians with renal disease was 4.05 mmol/L, while the mean total cholesterol to high-density lipoprotein ratio was 5.5. Whilst the total cholesterol of Aboriginal Australians with kidney disease was significantly lower than both PerioCardio participants (5.0 mmol/L) and Australian estimates (5.1 mmol/L), the total cholesterol to high-density lipoprotein ratio was significantly higher among Aboriginal Australians with kidney disease when compared to Australian estimates (3.7).

**Table 3:** Anthropometric and biomedical characteristics of Aboriginal Australians with kidney disease in Central Australia compared to PerioCardio participants and Australian population estimates

	Central Australian Aboriginal Australians with kidney disease mean (95% CI) (N = 102)	PerioCardio study mean (95% CI) (N = 273)	Australian population estimates (95% CI)^1,2^
Weight (kg)	81.41 (75.49, 87.32)	84.17 (81.62, 86.71)	79.40 (78.81, 79.99)
Body mass index^1^	29.20 (26.91, 31.48)	29.16 (28.29, 30.02)	28.10 (27.46, 28.75)
Waist to hip ratio	0.97 (0.94, 1.00)	0.94 (0.93, 0.95)	–
Sitting systolic BP (mmHg)^1^	143 (136, 151)^†^	125 (123, 127)	123 (122, 123)
Sitting diastolic BP (mmHg)^1^	85 (80, 89)^†^	80 (79, 81)	77 (77, 77)
Max. carotid intima-media thickness	0.80 (0.70, 0.90)	0.86 (0.82, 0.89)	–
HbA1c (mmol/mol)^2^	54.42 (46.04, 62.81)^†^	44.97 (42.95, 46.99)	36.20 (35.81, 36.59)
eGFR (mL/min/1.73m^2^)^2^	32.62 (17.79, 47.45)^†^	–	85.50 (85.30, 85.70)
Urine albumin	1,364.31 (24.91, 2,703.71)	41.92 (18.71, 65.13)	-
Urine creatinine	10.05 (5.95, 14.14)	7.92 (7.15, 8.69)	–
Urine albumin-creatinine ratio (mg/g)^2^	250.19 (19.86, 480.52)^†^	7.26 (3.74, 10.78)	2.2 (− 16.03, 20.43)
Total cholesterol (mmol/L)^2^	4.05 (3.66, 4.44)^†^	4.98 (4.85, 5.12)	5.1 (4.51, 5.69)
Triglycerides (mmol/L)^2^	2.43 (1.90, 2.96)	2.53 (2.30, 2.76)	1.3 (− 0.66, 3.26)
HDL cholesterol (mmol/L)^2^	0.82 (0.70, 0.94)^†^	1.04 (1.00, 1.08)	1.3 (0.52, 2.08)
LDL cholesterol (mmol/L)^2^	2.16 (1.86, 2.45)^†^	2.91 (2.78, 3.04)	3.1 (2.32, 3.88)
Total/HDL cholesterol ratio^2^	5.48 (4.69, 6.27)	5.22 (4.98, 5.45)	3.73 (2.95, 4.51)

^1^National Health Survey First Results 2017–2018, 4364.0.55.001 (participants 18 + years of age)

^2^Australian Health Survey: Biomedical Results for Chronic Diseases, 2011–12, 4364.0.55.005 (participants 18 + years of age)

Australian Health Survey 2017–2018: https://www.abs.gov.au/AUSSTATS/abs@.nsf/DetailsPage/4364.0.55.0012017-18?OpenDocument

95% CI calculator for mean: https://www.socscistatistics.com/confidenceinterval/default2.aspx SE converter for SD: https://handbook-5-1.cochrane.org/chapter_7/7_7_3_2_obtaining_standard_deviations_from_standard_errors_and.htm

^†^Denotes non-overlapping 95% confidence intervals

## Discussion

This investigation highlights stark differences in the oral health of Aboriginal Australians with kidney disease in terms of periodontal disease and dental caries when compared with estimates from both the general Australian population and against another sample of Aboriginal Australian adults from the Northern Territory. Of Aboriginal Australians with kidney disease who were screened, only 7.5% did not have moderate or severe periodontal disease, and the prevalence of those with severe periodontal disease was 20 times that reported for the general Australian population. While we expected periodontal disease to be more prevalent and more severe among people with kidney disease [23], the magnitude of poorer oral health among adults with CKD was striking.

Others have cited high prevalence of periodontal disease among CKD and ESKD patients. In Romania, Vessia and colleagues reported that among 101 hemodialysis patients with ESKD (mean age 53 years), estimates of periodontal disease, calculus index and bleeding on probing were on par with those reported in our study [24]. In a review of periodontal disease among patients receiving dialysis, Miyata and colleagues reported that periodontal diseases affected inflammation, the immune response, and nutritional status of patients on dialysis, with the severity of periodontal disease being significantly associated with CRP, albumin, IL-6 and TNF-α [25]. The authors demonstrated how dialysis exacerbates oral conditions via disruption of salivary characteristics (mainly pH and flow rate) which, in turn, contributes to severity of periodontal disease. They concluded that treatment and maintenance of oral health are important for quality of life, prevention of pathological conditions and prolongation of survival among dialysis patients.

It is important to discuss inequity in relation to access to preventive health care (including dental health care) in the context of our findings. Aboriginal Australians with kidney disease were older, resided in a remote location, had attained a lower level of formal education, were less likely to be employed, and more likely to be receiving government benefits as their main source of income when compared with other populations included in this study. Indeed, our group has previously reported the association between indicators of CKD and disadvantage [26], and there is a regional variation in CKD among the wider Australian population [27]. People with end-stage kidney disease require either haemodialysis, peritoneal dialysis or a kidney transplant to survive [28]. The majority of Aboriginal Australians with kidney disease in our sample undergo haemodialysis in thrice-weekly sessions lasting up to five hours. Under these circumstances, engaging in regular employment can be challenging due to co-existing health conditions, and thus financial support with government benefits was not unexpected. In a study involving 2,914 adults with moderate-to-severe CKD across 14 countries, Morton and colleagues showed how 32% were living in relative poverty, defined as having a household income < 50% of country median income, at study commencement. Amongst those not living in relative poverty at study commencement, 22% were living in poverty by study end 5 years later [29]. The authors concluded that progression of CKD is associated with increased odds of falling into poverty, with kidney transplantation being a potential mitigating factor against this. In relation to our findings, poverty among Aboriginal Australians with kidney disease likely contributes to the many challenges they may face in seeking and receiving timely dental care.

We also report differences in tooth loss among Aboriginal people of the Northern Territory with and without kidney disease. Tooth loss is the end-point of periodontal disease, equivalent to amputation in medicine. Consequently, loss of teeth eliminates the previous history of periodontal disease (and caries), but also serves to underestimate oral disease experience [30]. The extent of PPD, CAL and BOP findings presented in Table 2, which account for tooth loss, indicates that the number of areas throughout the mouths of Aboriginal Australians with kidney disease affected by periodontal disease is double that of the PerioCardio study participants. The NSAOH estimated in 2007 that approximately 30% of Aboriginal adults had periodontal disease [16]. Several observational studies involving Aboriginal people in urban and remote locations all posit that periodontal disease is much more common than in the non-Aboriginal Australian population [31, 32].

Cigarette smoking was attributed to 38.5 million cases of periodontal disease globally in 2015 [33]. Australian estimates from NSAOH reported that smoking contributed to 700,000 cases of ‘moderate’ or ‘severe’ periodontal disease [34]. The key mechanism by which cigarette smoking or tobacco chewing causes periodontal disease is by interfering with vascular and immunologic reactions, as well as by undermining the supportive functions and wound healing capabilities of the periodontal tissues [35]. The prevalence of cigarette smoking has steadily decreased in Australia in response to strict tobacco legislation and a raft of measures including marked increases in taxation and plain packaging laws [36]. This key public health policy has contributed to an 8.6% reduction in smoking rates among Aboriginal Australian adults from 50% in 2004 to 41.4% in 2015 [37]. In this light, the frequency of current smokers among Aboriginal Australians with kidney disease at 32.5% is lower than the latest Aboriginal Australian population estimate; however, exposure to tobacco in our sample was offset with almost 1-in-5 reporting they regularly chewed tobacco. This is consistent with previous Central Australian data and reflects differences between the Central Australia regions and Top End (hence between the Aboriginal Australians with kidney disease and PerioCardio study estimates) [38]. Whilst tobacco use is a major cardiovascular disease (CVD) risk factor, and CVD a major co-morbid condition of adults with CKD, we are unaware of reports describing successful smoking cessation among Aboriginal adults with CVD and/or CKD.

Untreated caries is a major contributor to dental pain affecting mastication (chewing), which directly affects dietary choices, malnutrition risk and quality of life [39]. Periodontal disease is not usually painful unless abscesses develop. However, periodontal disease does impact on quality of life through loss of function and aesthetics [40]. In this study, there was a higher amount of untreated caries, and lower number of filled teeth among Aboriginal Australians with kidney disease compared with both PerioCardio and NSAOH participants. Both indicators (caries and filled teeth) may reflect individual-level dental seeking behaviours or oral health services accessibility. Combined with the higher mean number of missing teeth, we speculate that among this sample of Aboriginal Australians with kidney disease there was a high level of oral health seeking behaviour for management (tooth extraction), which is consistent with other studies involving Aboriginal Australians [41–43]. It is worth noting that inadequate access to health services for Aboriginal Australians with kidney disease is not limited to dental services. Morton and colleagues demonstrated in a systematic review among adults with moderate-to-severe CKD, that socially disadvantaged pre-dialysis and dialysis patients experience poorer access to specialist cardiovascular health services, higher rates of cardiovascular events and mortality than their more advantaged counterparts [44].

The most significant relevant bias relates to the different designs of the included studies: the study involving Aboriginal Australians with kidney disease and the PerioCardio study were both convenience studies, while the four other datasets were representative, population-level surveys (with weighted estimates) or the Census. Potential participants for both the study involving Aboriginal Australians with kidney disease and the PerioCardio study were invited to be involved in a periodontal health project, which clearly caused selection bias with the samples thus not being representative. In both studies, there was no dental pre-screening done by those who referred potential participants to the study team. There have not been any truly population-representative studies reporting oral health and prevalence of periodontal disease among the Aboriginal Australian population, especially the population with kidney disease. We recommend additional research to report this.

In this observational study, we report poorer oral health status of Aboriginal Australians with kidney disease than the general Australian population, but also compared with other Aboriginal Australian adults living in the Northern Territory. Because of the profound health impacts and reduced quality of life associated with untreated periodontal disease and dental caries, the findings reinforce the importance of making and keeping oral health a priority action area for all people with kidney disease.

## Conclusions

Aboriginal Australians with kidney disease exhibited more indicators of poorer oral health than both the general Australian population and a general Aboriginal population from Australia’s Northern Territory. It is imperative that management of oral health among Aboriginal Australians with kidney disease be included as part of their ongoing medical care.

## Data Availability

Available from corresponding author upon request.

## Abbreviations

NSAOH: National Survey of Adult Oral Health
DMFT: decayed, missing and filled teeth
CKD: chronic kidney disease
ESKD: end-stage kidney disease
NHS: National Health Survey
AHS: Australian Health Survey
PPD: periodontal pocket depth
CAL: clinical attachment loss
BOP: bleeding on probing
BMI: body mass index
CI: confidence interval
SD: standard deviation
se: standard error
HbA1c: glycosylated haemoglobin
CRP: C-reactive protein
IL-6: InterLeukin 6
TNF: tumour necrosis factor
CVD: cardio vascular disease
